# Cultural adaption and validation of the Explanatory Model Interview Catalogue–Community Stigma Scale in the assessment of public stigma related to schistosomiasis in lakeshore areas of Mwanza region, Tanzania

**DOI:** 10.1371/journal.pntd.0011534

**Published:** 2023-08-14

**Authors:** Laura Klinker, Anne Boeckler, Saskia Kreibich, Humphrey Mazigo

**Affiliations:** 1 Department of Psychology, Julius-Maximilian University Wuerzburg, Wuerzburg, Germany; 2 Department of medical social projects, DAHW German Leprosy and Tuberculosis Relief Association, Wuerzburg, Germany; 3 Department of Parasitology and Entomology, Weill Bugando School of Medicine, Catholic University of Health and Allied Sciences, Mwanza, Tanzania; George Washington University School of Medicine and Health Sciences, UNITED STATES

## Abstract

**Background:**

Previous qualitative studies on attitudes towards schistosomiasis demonstrated inconclusive results on the extent of stigma towards schistosomiasis in endemic communities around the world. The Explanatory Model Interview Catalogue–Community Stigma Scale (EMIC-CSS) has been used and validated for the assessment of public stigma across numerous countries in various health conditions. This study tested the performance of the scale in the context of stigma related to schistosomiasis in twelve communities in the three districts of Magu, Nyamagana and Ilemela in Mwanza region, Tanzania.

**Methodology/Principal findings:**

The 15-item-version of the EMC-CSS was first translated to Kiswahili language. The translation was discussed within the research team to retain the meaning of the items and implement cultural adaptations. Validation of the adapted EMIC-CSS scale was conducted following the framework of Herdman and Fox- Rushby. A pilot study with 41 participants from two communities provided the basis for testing the performance of each item and assessing the semantic and operational equivalence of the scales. In addition, eight qualitative focus group discussions (FGDs) were conducted to evaluate the conceptional equivalence of the EMIC-CSS. Finally, the performance of the adjusted scale was tested on 200 participants with a 50:50 male-female ratio from ten communities. The mean score of the EMIC-CSS M = 8.35 (SD = 6.63) shows clear indications for public stigma towards schistosomiasis. The EMIC-CSS demonstrated a good internal consistency with Cronbach’s alpha α *=* .*857* and no floor and ceiling effects.

**Conclusion/Significance:**

The results demonstrate that the EMIC-CSS is a useful instrument in assessing public stigma towards schistosomiasis and allow a clear recommendation of the EMIC-CSS for schistosomiasis in the Tanzanian culture. However, future studies are additionally recommended to address specific aspects and forms of the disease and how they contribute to the development of stigma towards schistosomiasis.

## Author summary

Schistosomiasis is a common disease in many lakeshore areas across Africa, especially in communities of a lower socio-economic status with inadequate access to clean water and hygienic sanitary facilities. The disease is caused by parasitic worms transmitted by fresh water snails in infested water bodies and can be lethal, if the infection and related chronic morbidities aren’t timely treated. Available evidence indicates that people suffering from schistosomiasis experience stigmatization from community members and healthcare providers when seeking health care services. However, this has not been systematically examined. In the present study the EMIC-CSS questionnaire was adapted and validated according to the framework of Herdman and Fox-Rushby in order to investigate the extent of stigmatization due to schistosomiasis in Mwanza region, Tanzania, where the disease is highly endemic. The scale was successfully validated and the results indicate that schistosomiasis-related stigma is indeed common in communities living along the Lake Victoria in Mwanza region.

The findings of this study can improve knowledge and create awareness about the existence of community-related stigma towards persons infected with schistosomiasis and, as a result, can be used to contribute to the control and elimination of schistosomiasis in Tanzania.

## Introduction

The neglected tropical disease (NTD) schistosomiasis, also known as snail fever and bilharziasis, is a water-borne parasitic disease caused by parasitic flatworms [1]. Infection occurs as a result of contact with fresh water contaminated with parasites released from infected fresh water snails. The causative agents of schistosomiasis are blood trematodes–with two different species types being present in Sub-Saharan-Africa: *Schistosoma mansoni* causing intestinal schistosomiasis and *Schistosoma haematobium* resulting in urogenital schistosomiasis which is characterized by haematuria (blood in urine), pain during urination and lower abdominal pain [2]. Schistosomiasis is considered a disease associated with poverty, as it predominantly affects rural communities characterized by low socio-economic status that lack adequate access to clean water and proper sanitation [1]. Therefore marginalized, poverty-affected communities living in schistosomiasis endemic areas are considered vulnerable to an infection and, as a result, prone to its disease-related stigma [3–6].

Stigma related to a health condition refers to any negative attitude towards persons suffering from a health condition, that can severely impact the quality of life of the persons stigmatized [3,7,8]. Health-related stigma is often characterized by social disqualification processes such as labeling, stereotyping, status loss and discrimination [9]. Even though the health conditions may vary from mental health problems to neglected tropical diseases (NTDs), the extent and forms of stigmatizing are largely similar [3,10]. Stigma towards neglected tropical diseases has received increasing attention in the last two decades [3,4,11–13]. Not only physical impairments and disfigurements, as a result of an infection with NTDs, are associated with stigma [3–6]. The whole intersection of stigma based on socio-economic status, ethnicity and a health condition amongst others can result in an enormous burden for those affected [3,14].

Previous studies assessing attitudes and perceptions towards schistosomiasis have rarely addressed stigma towards schistosomiasis directly. However, several forms of stigma associated with the disease have been reported and suggest that schistosomiasis is considered a *shameful* disease [6,15–18]. Intrinsic negative attitudes towards the disease within the person affected, known as *internalized stigma* [19], were expressed in the form of s*hame*, *embarrassment* [15,17,18,20] and *the fear of being a financial burden to the family* [15]. *Anticipated stigma* outlines the expectation of stigma by the affected person [19] and was apparent in the form of *fear of disclosure and fear of stigma* [6,15,17,18]. The expression of discrimination due to schistosomiasis, called *enacted stigma*, [19] was described as a*voidance*, *negative attitudes*, *labelling* and *making fun of schistosomiasis patients* [15,17,18,20]. However, reports of schistosomiasis being considered shameful contradicts research describing schistosomiasis as being perceived as a *normal* disease [16,21,22]. Two qualitative studies addressing stigma related to female genital schistosomiasis have described stigmatizing attitudes similar to experiences when having a sexually transmitted disease [23,24]. Stigma towards schistosomiasis has not yet been investigated by means of a quantitative approach neither in Tanzania nor in another country with endemic communities. In order to understand the extent, the forms and the consequences of schistosomiasis-related stigma a standardized tool is needed. Assuming a generic concept of health-related stigma, the use of similar stigma assessment tools across different health conditions and countries is recommended [8,10]. The Explanatory Model Interview Catalogue–Community Stigma Scale is one of the most frequently used and validated questionnaires for the assessment of public stigma in health conditions [10,12,25–28].

The original EMIC used a mixed methods approach including quantitative and qualitative parts in order to examine the experience of illness of affected people [29]. Recent research largely focused on the quantitative part of the catalogue, the Community Stigma Scale, which was adapted to enable measurement of stigmatizing attitudes and behaviors in the communities in which it was applied [10]. As a result, multiple versions of the scale exist with the number of items of the scale varying between 8 to 25 [10]. The response-scale contains four options ‘Yes’ [2]; ‘Possibly’ [1]; ‘No’ (0) and ‘Don’t know’ (0) [10]. Stigma related to various NTDs, mental health and other conditions has been examined using the EMIC-CSS questionnaire in Nigeria, Cameroon, Ghana, Uganda, India, Nepal, Indonesia and Thailand. [10,12,20,25–28,30]. For the 15-item-version a mean sum score of 8 and higher is perceived to be a meaningful indication of stigma for health-related stigma [25]. The 15-item-scale was adapted for the context of leprosy [27] in Indonesia following the framework for cross-cultural equivalence of Herdman and Fox-Rushby [31] and recommendations of Terwee et al. [32] and Stevelink and van Brakel [33]. The cultural equivalence framework of Herdman and Fox-Rushby comprises conceptual, item, semantic, operational and measurement equivalence and is displayed in Table 1 [31].

**Table 1:** Overview of conceptual, item, semantic, operational and measurement equivalence.

Form of equivalence	Description	Recommended analysis
**Conceptual**	Conceptual equivalence ensures that the concepts, e.g. of stigma, are equally useful, relevant and accepted in different cultures.	qualitative analysis via expert interviews, focus group discussions, literature analysis
**Item**	Item equivalence is assessed to ensure that the items of the adapted scale intended for use in a different culture, are equally relevant and accepted as the original.	expert judgement/discussion of the items through people of the target culture
**Semantic**	Semantic equivalence is assessed to ensure that the translated items of the adapted scale, intended for use in a different culture, have the same meaning as the original	paraphrasing of the items, discussion of the different meanings, forward and backward translation
**Operational**	Operational equivalence is assessed to ensure implementation of the adapted scale in the new setting can be achieved using a similar format, response scales and instructions.	literature review on instrument use in target culture, consideration of literacy
**Measurement**	Measurement equivalence is assessed to ensure that the reliability of the adapted EMIC-CSS scale is equal compared to the original scale. The quality of the psychometric properties is assessed according to Terwee et al. [32]: analysis of content validity, internal consistency, criterion validity, construct validity, reproducibility, longitudinal validity, responsiveness, floor and ceiling effects, interpretability	determined according to the recommendations of Terwee et al. [32] Cronbach’s alpha for internal consistency, intraclass correlation coefficient, test of responsiveness: paired t-test, effect size statistic, responsiveness statistic, tests of construct validity, factor analysis

If all categories of the cultural equivalence framework are properly reflected in the scale, cultural equivalence is achieved and the scale can be considered validated in the new culture [31]. In their large study of cultural adaption and validation (N > 200), Peters et al. [27] tested on convergent validity of the EMC-CSS with the Social Distance Scale of r = 0.41 and showed Cronbach’s alpha α = 0.83 and a retest reliability coefficient of 0.84. The process of validating the 15-item version of the EMIC-CSS across various cultures and health conditions has demonstrated different understandings of the items, proposed new response scales and has shown different sub-components in factor analysis [27,34]. These findings underline the importance of cultural validation prior to widespread use of health questionnaires in diverse health and cultural settings. In this study, the English version of the EMIC-CSS for the context of leprosy was adapted to the context of schistosomiasis and translated to Kiswahili. In the Tanzanian culture, the EMIC-CSS has been used in Kiswahili language for the assessment of stigma related to albinism [34,35]. A self-assessment version of the scale was tested and adapted on cultural equivalence [35]. This Kiswahili version was not yet accessible when adapting and validating for schistosomiasis.

In this study, the 15-item version of the EMIC-CSS with the above mentioned four-option scale was used. It was supplemented with demographic items and a questionnaire on knowledge and perceptions on schistosomiasis. Yet, due to the focus on the validation of the EMIC-CSS, the additional survey parts will not be addressed in this article.

## Methods

### Ethics statement

The study received ethical approval from the Lake Zone Institutional Review Board of the National Institute for Medical Research, Tanzania (Certificate number: MR/53/100/570). Region and district authorities provided formal approval for implementation in their respective territories. Participation was fully on a voluntary basis. Informed written consent and assent were obtained from all study participants prior to inclusion into the study. Consent and assent forms were read aloud to illiterate participants and their finger print was used as signature. All participants were encouraged to ask questions and all mentioned concerns were addressed. Anonymity of the study participants and confidentiality of information was maintained throughout the course of implementation, data entry, analysis and publication.

### Study composition

Validation of the cultural adaptation of the EMIC-CSS was conducted according to the cultural equivalence framework by Herdman and Fox-Rushby and followed a quantitative and qualitative approach [10,27,31,34,36–38].

First a pilot run of the EMIC-CSS (N = 41) was implemented for quantitative analysis, accompanied by focus group discussions (N = 81). The focus group discussions aimed to assess conceptual equivalence via qualitative analysis. The insights of the quantitative and qualitative investigation were used to adapt the translated version of the EMIC-CSS.

Consequently, a cross-sectional study (N = 200) was conducted to assess the performance of the adapted EMIC-CSS and to measure the extent of stigma towards schistosomiasis in the communities which were selected.

### Design of the cultural validation process

The adjusted version of the EMIC-CSS was applied and validated following the cultural equivalence framework of Herdman and Fox-Rushby [31]. Specifically, the aim was to assure conceptual, item, semantic, operational and measurement equivalence of the scale. Table 2 provides an overview of the taken steps according to the framework of cultural equivalence of Herdman and Fox-Rushby [31].

**Table 2:** Overview of the strategies to test for conceptual, item, semantic, operational and measurement equivalence.

Form of equivalence [31]	Methods
**Conceptual**	1. Qualitative and quantitative pilot 1a. Qualitative analysis of forms of stigma related to schistosomiasis in focus group discussions in 8 communities (N = 81) (3 female, 3 male, 2 mixed FGDs): qualitative assessment of perceptions and attitudes towards schistosomiasis/schistosomiasis patients, effects in quality of life/social life due to the disease, coding and clustering of the results, comparison with the seven aspects of the EMIC-CSS 1b. Pilot study of the translated EMIC-CSS (N = 41) 2. Main implementation of the adapted EMIC-CSS in ten communities (N = 200)
**Item**	1. Discussion of items in the research group with interdisciplinary backgrounds (medicine & public health, study nurse, social sciences, psychology) with particular expertise on schistosomiasis and Tanzanian culture: analysis of relevance and acceptance of the items in the target culture, Tanzania 2. Evaluation of experiences of the interviewer in the pilot study
**Semantic**	1. Translation and discussion in the research team of the target culture and with people with heterogeneous knowledge on schistosomiasis and stigma: interdisciplinary backgrounds (medicine & public health, study nurse, social sciences) 2. Pilot implementation: evaluation of the interview process (e.g. possible misunderstandings) and the results (mean, outliers of individual items) 3. Translational adaptions of the EMIC-CSS after the pilot implementation 4. Backtranslation of the adapted EMIC-CSS from Kiswahili language to English in order to ensure the meaning of the items in the target language 5. Comparison with the Kiswahili version of EMIC-CSS assessing stigma related to albinism in Tanzania [34]
**Operational**	1. Instrument use in target population considering literacy 2. Pilot and main implementation: evaluation of the interview process
**Measurement**	1. Frequencies Assessment of floor and ceiling effects of the items in the adapted EMIC-CSS scale 2. Internal consistency and item selectivity 2a. Cronbach’s alpha 2b. Item-total correlations 3. Explanatory factor analysis Varimax rotation 4. Differences between the mean scores of interviewers: Kruskal-Wallis-test for independent samples

### Study setting

The study was conducted in villages along the shoreline of the Lake Victoria at Magu, Nyamagana and Ilemela districts of Mwanza region, Tanzania. Villages known to be endemic for intestinal and urogenital schistosomiasis were selected [39,40]. Because of their proximity to Lake Victoria, high rates of poverty and everyday activities involving frequent contact with lake water due to the lack of adequate water, hygiene and sanitation infrastructure, the villages are at high risk for schistosomiasis [39,40]. The pilot was conducted in Ijinga and Lugeye village which belong to Magu district. Four of the focus group discussions were conducted in Ilemela (Kayenze Iseni, Igalagala, Jamimbi Lutongo, Kabangaja) and four of them in Nyamagana (Mihama, Butuja, Bwiru Ziwani, Kigoto). The implementation of the EMIC-CSS in the main study took place in ten different communities: five in Ilemela district (Kayenze, Kayenze ndogo, Igalagala, Jamimbi and Lutongo) and five in Nyamagana district (Mihama, Butuja, Bwiru ziwani, Igombe, Kigoto).

### Sampling and data collection procedures

A total of 322 people from selected lakeshore communities participated. Communities in which contact with people who have or have had schistosomiasis is highly likely were chosen. Inclusion and exclusion criteria were identical for all study components.

Inclusion criteria:

- Adult age (> = 18 years)
- consent to participate in the study
- resident of the target communities

Exclusion criteria:

- signs and symptoms for health concerns
- persons who cannot express themselves (e.g. due to speech or cognitive impediment)
- not sufficiently fluent in Kiswahili language

All interviews and focus group discussions were conducted by a local research team.

### a) The survey: Demographics, EMIC-CSS, knowledge and perceptions

The 15-item-version used by Van Brakel et al. and Peters et al. addresses seven aspects of stigma; concealment, process of discrediting, shame and embarrassment, avoidance/keeping distance/isolation, problems with getting married or ongoing marriage, problems for family or other people and problems with work [27,33]. In order to gain a better understanding of the knowledge about and the attitudes towards schistosomiasis, the EMIC-CSS was embedded in a survey considering demographics and a knowledge and perceptions questionnaire.

### b) Pilot implementation of the EMIC-CSS

In a first step, the 15-item version of the EMIC-CSS was translated into Kiswahili language and proofread by a sociomedical research team.

The pilot study of the EMIC-CSS (N = 41; 30 female and 11 male participants) and the concluding demographics, knowledge and perceptions questionnaire were conducted in Ijinga and Lugeye. These two villages were considered to give a broad picture on perceptions related to schistosomiasis due to their different levels of knowledge and education on schistosomiasis. Lugeye has been largely neglected in schistosomiasis control measures, whereas Ijinga is an island where several schistosomiasis campaigns have been conducted in recent years [41,42]. Contact to the participants of this study was established by community drug distributors or community leaders by convenience sampling. Participants were met at their homes. The assessment was operated in form of interviews by local female and male interviewers speaking Kiswahili language and at least a minimum of Kisukuma language as many interviewees had Kisukuma language as mother tongue. The gender of interviewer and interviewee was not matched. Translational challenges and misunderstandings of the pilot study were discussed afterwards with the sociomedical research group.

### c) Qualitative data collection

As part of the pilot study, eight focus group discussions (FGDs) were conducted in eight different communities to inform about possible needs for adaptations of the main quantitative survey. Participants were systematically selected and contacted by local drug distributors and community leaders in order to form a group that represents the community in terms of age, occupation, housing and education. The focus group discussions were conducted in designated spaces of community centers. Each FGD was conducted by one facilitator and one observer. To minimize gender effects, the FGDs with female participants were led by a female research team, the FGDs with male participants by a male research team and the FGDs with both male and female participants by a mixed research team. In total, 41 women and 40 men participated in the FGDs: ten or eleven participants per FGD. The discussions were conducted in Kiswahili language. As some people in the communities may mainly speak Kisukuma language, the language of the Sukuma tribe, there was at least one person speaking Kisukuma language in each facilitator team. Hence, people speaking little Kiswahili were included in the FGDs.

The guiding questions for the FGDs were developed based on recent literature on perceptions, attitudes and stigma towards schistosomiasis. The guiding questions comprised the following subjects: *perception of schistosomiasis compared to other diseases*, *thoughts/feelings about schistosomiasis*, *knowledge about schistosomiasis*, *talking about schistosomiasis in the community*, *risk perception of being infected (Who is at particular risk to get schistosomiasis*?*)* and *coping with schistosomiasis infection*. Schistosomiasis-related stigma as a relevant concept in Tanzanian culture was addressed with questions about how people talk about schistosomiasis or schistosomiasis patients; which thoughts and emotions occur when thinking of schistosomiasis; whether schistosomiasis is perceived as a serious problem in the community; whether thoughts and concerns about the disease could affect daily life, which people may be more or less vulnerable to schistosomiasis.

### d) Main implementation of the survey: adapted EMIC-CSS, demographics and the knowledge and perceptions questionnaire

For the main implementation of the EMIC-CSS, 240 participants were assessed in ten different communities. In addressing ten different communities, a broader range of occupations due to geographical patterns was covered in the sample to enhance representativity for Mwanza region. The data collection was conducted throughout the whole day in order to allow different people to participate independent of their duties. Participants were contacted through community drug distributors, health workers or community leaders following convenience sampling. The interviews were held at the participants’ homes. As the community questionnaire contained sensitive topics such as sexual performance etc., female interviewers were assigned to female participants and vice versa. Unfortunately, 40 of these community questionnaire interviews were conducted with mismatched interviewers: female interviewers interviewed male participants and male interviewers assessed female participants. These data were excluded from the analysis. As many of the participants belong to the Wasukuma tribe and speak Kisukuma as mother tongue, many of the interviewers were capable of speaking at least basic Kisukuma language being able to translate difficult words if needed. People who did not speak Kiswahili at all were excluded from the survey assessment of the EMIC-CSS.

In the main implementation, the EMIC-CSS was complemented by demographic questions and a knowledge questionnaire containing important factors regarding schistosomiasis-related stigma identified during the FGDs and in previous literature. As this article aims to describe the assessment of the EMIC-CSS, the results of the knowledge questionnaire are not reported here.

#### Data management and analysis

The data of the pilot and the main implementation of the EMIC-CSS were assessed paper-based and then digitalized by the research team. In the main implementation, data of 40 participants with mismatched interviewers (female interviewers interviewing male participants and vice versa) were excluded from the data analysis.

The focus group discussions were recorded simultaneously by a Sony PX470 voice recorder and an Apple Iphone 5s with the pre-installed recording app. The transcriptions were conducted by two research assistants per FGD; one person transcribed the FGD and the other person did the proofreading. The translation process from Kiswahili language to English was similar—one research assistant translated and the second research assistant was responsible for proofreading.

The statistical analysis of all quantitative data was conducted using the IBM SPSS Statistical software package 21.

The internal consistency of the EMIC-CSS scale was assessed by means of calculating the item-total correlations and the Cronbach’s alpha. Consequently, frequencies of all variables of the EMIC-CSS scale were calculated to assess the collected data on floor and ceiling effects. A principal component analysis with varimax rotation was conducted to investigate the factor structure of the EMIC-CSS. Finally, differences between the mean scores of the interviewers were tested. The influence of individual interviewers on participants’ responses was analyzed by means of a Kruskal-Wallis test for independent samples, as the four interviewers talked to different numbers of participants, leading to unequal sample sizes. The Kruskal-Wallis-test was conducted with the interviewer as factor and the sum score of the EMIC-CSS (EMIC-Sum Score) as dependent variable. Post-hoc Dunn Bonferroni tests were used in order to compare all individual interviewers.

Qualitative data were analyzed using VERBI MAXQDA 2018.2. The data were first coded and clustered to identify potential stigmatizing attitudes and perceptions towards schistosomiasis. These were consequently compared to the findings of the EMIC-CSS scale. In order to avoid false-positive findings, not only confirming statements but also discrepant statements were searched for in the qualitative analysis.

## Results

### Sociodemographic characteristics

The mean age in the pilot was 37 years (SD = 16); in the focus group discussions 44 years (SD = 13) and in the main implementation 41 years (SD = 14). The data of 97 female and 103 male participants were included in the main implementation, 30 female and 11 male participants in the pilot study; 40 male and 41 female participants in the focus group discussions. The majority of the participants were from Sukuma ethnicity (55.28%), were married (75.16%) and were Christians (77.33%). Characteristics of participants of the pilot study, focus group discussions and main implementation of the EMIC-CSS are depicted in Table 3.

**Table 3:** Sociodemographic characteristics of participants in the pilot study, the FGDs and the main study.

Variables		Pilot study (N = 41)	Focus group discussions (N = 81)	Main study (N = 200)
**Sex** ** **	Female (%)	30 (73.2%)	41 (50.6%)	97 (48.4%)
	Male (%)	11 (26.8%)	40 (49.4%)	103 (51.6%)
**Age**	Mean (SD)	37 (15.6)	44 (13.0)	41 (14.0)
**Household number**	Mean (SD)	7.41 (3.6)	N/A	6.36 (2.9)
**Ethnic group** ** **	Sukuma	40 (97.6%)	47 (58%)	91 (45.5%)
	Msumbwa	1 (2.4%)		
	Jita		9 (11.1%)	28 (14.0%)
	Muha		5 (6.1%)	14 (7.0%)
	Kerewe		7 (8.6%)	19 (9.5%)
	Haya			
	Mkwaya			7 (3.5%)
	Other		13 (16.0%)	34 (17.0%)
**Marital status** ** **	Married	29 (70.1%)	66 (81.5%)	147 (73.5%)
	Consensual union	8 (19.5%)		
	Single		8 (9.9%)	23 (11.5%)
	Widowed	2 (4.9%)	5 (6.3%)	19 (9.5%)
	Divorced			6 (3.0%)
	Separated			4 (2.0%)
	Other	2 (4.9%)	2 (2.5%)	
**Religion** ** **	Christian	23 (56.1%)	69 (85.2%)	157 (78.5%)
	Muslim		12 (15.0%)	24 (12.0%)
	Pagan	11 (26.8%)		7 (3.5%)
	Other	7 (17.1%)		12 (6.0%)
**Educational level** ** **	Illiterate	18 (43.9%)		55 (27.5%)
	Primary	21 (51.2%)	62 (76.5%)	103 (51.5%)
	Not completed Secondary			6 (3.0%)
	Secondary		10 (12.3%)	28 (14.0%)
	College	1 (2.4%)		7 (3.5%)
	Education of higher learning institutions			1 (0.5%)
	Other	1 (2.4%)	9 (11.1%)	
**Occupation** ** **	Peasants	38 (92.7%)	48 (59.3%)	91 (45.5%)
	Fishermen/women			19 (9.5%)
	Small businesses		9 (11.1%)	59 (29.5%)
	Drivers		3 (3.7%)	
	Housewives			12 (6.0%)
	Other	3 (7.3%)	9 (11.1%)	10 (5.0%)

SD refers to the standard deviance. The household number describes the number of people living in the household of the participants. The educational level is assessed by the graduation level of primary or secondary school, college or higher learning institutions. Primary school ends after seven years (Standard I–VII) whereas Secondary school ends after four years of school (Form 1–4).

### Descriptive data

Main survey implementation of the EMIC-CSS resulted in mean sum scores of M = 7.24 (SD = 5.66) for women and M = 9.28 (SD = 7.24) for men. Thus, the mean sum score of the EMIC-CSS in the pilot and in the main study exceeds the score of 8 which is perceived to be a meaningful indication of stigma for health-related stigma [25]. The range of each item is 2.0 (0 = no; 1 = possibly; 2 = yes) and describes the degree of agreement per item. With 15 items total, the maximum sum score possible is 30. 43% (86 participants) reached the defined cutoff-value and thus reported stigma [25], 45% (90 participants) did not reach the cut-off value. Participants answering single items with “I don’t know” (12%) were excluded when considering the EMIC-CSS sum score. The overall mean sum score of M = 8.35 (SD = 6.63) indicates the existence of stigmatizing attitudes and perceptions among participants. The mean sum scores and matching standard deviations of each item of the adapted EMIC-CSS of the pilot study and main survey are displayed in Table 4.

**Table 4:** Mean scores and standard deviations of pilot study and main study.

	Pilot study (N = 41)		Main study (N = 200)	
Item	M	SD	M	SD
**Sum score**	10.08	6.32	8.35	6.63
*1. Would a person with schistosomiasis keep others from knowing*, *if possible*?	0.39	0.77	0.42	0.74
2. *If anyone in your family suffers from schistosomiasis*, *does it impair your worth*?	1.24	0.97	0.63	0.91
3. *In your community*, *does schistosomiasis cause shame or embarrassment*?	1.32	0.96	0.91	0.98
4. *Would others think of a person with schistosomiasis being worthless?*	0.66	0.88	0.35	0.72
5. *Would knowing that someone has schistosomiasis have an adverse effect on others*?	0.78	0.96	0.82	0.98
6. *Would other people in your community avoid a person affected by schistosomiasis*?	0.32	0.72	0.26	0.66
7. *Would others refuse to visit the home of a person affected by schistosomiasis*?	0.34	0.73	0.22	0.60
8. *Would people in your village think that families with schistosomiasis patients are impaired in their worth?*	0.22	0.61	0.30	0.68
9. *Would schistosomiasis cause problems for the family*?	1.12	0.98	0.99	1.00
10. *Would a family have concern about disclosure if one of their members had schistosomiasis*?	0.46	0.81	0.51	0.85
11. *Would schistosomiasis be a problem for a person to get married*?	0.98	0.96	0.47	0.83
12. *Would schistosomiasis cause problems in an on-going marriage*?	0.63	0.86	0.58	0.90
13. *Would having schistosomiasis cause a problem for a relative of that person to get married*?	0.53	0.85	0.42	0.80
14. *Would having schistosomiasis cause difficulty for a person to find work*?	0.41	0.81	0.26	0.66
15. *Would people buy food from a person affected by schistosomiasis*?	0.71	0.93	1.41	0.89

### Results of the cultural validation process

The results of the analysis of the five aspects of cultural equivalence (as described in Table 2) are displayed in the following.

#### Conceptual equivalence

Coded and clustered topics discussed during the FGDs confirm the relevance of aspects addressed in the adapted EMIC-CSS. The topic categories of the EMIC-CSS derived from the validation study of Peters et al. [27] were met in the coded and clustered subjects. Statements made in the FGDs supporting and conflicting the different aspects of the EMIC-CSS are provided in Table 5. Problems in marriage and therefore the aspect of concealment were addressed repeatedly, even though schistosomiasis was also described as a “normal disease” several times.

**Table 5:** Match of topics of the EMIC-CSS with addressed aspects in the FGDs.

Topics of the EMIC-CSS [27]	Confirming data of the FGDs	Conflicting data of the FGDs
Concealment	*“Even if he has been tested […] it is his secret*.*”* (FGD_02_Male Igalagala_Ilemela l. 1291) *“I don’t think it will be easy to tell your neighbor that I am suffering from schistosomiasis”* (FGD_06_Female Butuja, l. 2349). *“The two of us are the only ones that will know*.*”* (FGD_03_Female: Chamimbi, l. 2416) Difficulty to tell own husband: *“She cannot tell the husband*, *depends with the kind of the relationship they have*, *how close they are*.*”* (FGD_07_Male_ Bwiru ziwani, l. 2068)	*“I will be free to talk [about schistosomiasis*.*]”* (FGD_03_Female_Chamimbi, l.1788)
Process of discrediting	*“Even if you had a good health you will start losing some weight and that’s when people will start talking bad about you”* (FGD_03_ Female Chamimbi, l. 1054) *“[Schistosomiasis weakens your health] That’s why your community will think negative of you or otherwise about your type of disease”* (FGD_08_ Mixed Kigoto l. 395)	*“[…] honestly*, *I have never seen that [discrimination of a patient] here*.*”* (FGD_07_Male Bwiru Ziwani, l. 1229)
Shame and embarrassment	*“Ee it’s like one feels ashamed of telling someone that I have been tested and found infected*.*”* (FGD_06_Female_Butuja, l. 2354) symptom of urinating blood perceived as embarrassing: *“[…] if they found out that you were urinating blood*, *even if you had a woman in the community*, *you would still be embarrassed”* (FGD_05_Male Mihama, l.751)	*“They take it as a normal disease” (FGD_01_Female Kayenze*, l. 1569*)*
Avoidance/ taking distance/ isolation	*“This schistosomiasis disease ok*, *eeh when a patient is discovered that he has schistosomiasis–even if he is a child*, *other children will start isolating him”* FGD_04_Mixed Kabangaja, l. 348). *“Yes*, *there is exclusion because when you have schistosomiasis your soul and body will be weak”* (FGD_03_ Female_Chamimbi l. 918) Discrimination due to confusion of schistosomiasis with HIV/aids: *“They will discriminate you without knowing you have another disease”* (FGD_03_ Female_Chamimbi, l. 954)	*“[…] honestly*, *I have never seen that [discrimination of a patient] here*.*”* (FGD_07_Male Bwiru Ziwani, l. 1229) *“My feelings for a sick person with schistosomiasis are that you see him as common person like others with diseases*.” (FGD_02_Male_Igalagala, l. 1373). "*About the schistosomiasis disease*, *the community does not stigmatize*, *no patient can be isolated in the community but only a person with AIDS*” (FGD_05_male Mihama, l. 905)
Problems with getting married or ongoing marriage	*“There is the possibility of marriage to break up*.*”* (FGD_04_Mixed Kabangaja, l. 427) *“First will feel that his/her partner is cheating*.*”* (FGD_04_Mixed Kabangaja, l. 430) *“It reduces and it can cause marriage separation*.*”* (FGD_04_Mixed Kabangaja, l. 925) *“It affects the reproductive system then*, *how will you not be affected by that*?*”* (FGD_07_Male Bwiru Ziwani, l. 607). *“The relationship between a woman and a man collapses because if Schistosomiasis is at critical stage the man’s waist efficiency is reduced*.*”*(FGD_05_Male_Mihama, l.62)	
Problems for family or other people	Problems for the family mainly due to economic situation: “*My life condition will be very poor if I lay in bed not being able to wake up and go to work for my family*.*”* (FGD_03_female Chamimbi, l. 902) *“If you miss the needs*, *you can be faced by hunger and you can get a sick family member*, *what can you have for treatment because disease problems may happen*. *Let’s say the big problems is not having money for your living at the time”* (FGD_02_Male_Igalagala, l.287)	
Problems with work	“*That makes him loose the energy in his working capability […] he has become dependent”* (FGD_07_Male Bwiru Ziwani, l.1241) “*Concerning the issue of economy when you have been already affected by schistosomiasis you become weak and lose confidence in yourself”* FGD_08_Mixed Kigoto).	

#### Item equivalence

The items containing the expression “thinking less of oneself” (EMIC2, EMIC4 and EMIC8) lead to misunderstandings in the pilot study described by the interviewers. The misunderstandings were reflected in false-positive responses, i.e. shown in the second highest mean score for EMIC2 with M = 1.24 (*SD* = 0.97). These misunderstandings raised the question whether these items are culturally relevant, useful and accepted. After correcting translational misunderstandings, as described in the next paragraph *semantic equivalence*, the items were understood and accepted. The relevance of the items and their contribution to the scale can also be seen in the item-total-statistics. These are displayed under the measurement equivalence.

#### Semantic equivalence

In expert discussions with the interviewers after the pilot run, some misunderstandings could be identified that will be outlined in some detail. First, for the expression “*thinking less of oneself*” (EMIC2, EMIC4 and EMIC8) there is no equivalent expression in Kiswahili. Thus, the term was occasionally falsely understood as “*thinking less often of someone*” in the items EMIC4 and EMIC8. In item EMIC2, instead of the actual meaning “*If a member of your family had schistosomiasis*, *would you think less of yourself*?” the item was expressed the following way: *“Would you feel bad/sorry*, *if somebody of your family had schistosomiasis*?” leading to the above-reported false-positive answers in the pilot run. Accordingly, questions with the concept of “thinking less of oneself” were translated into

- *If anyone in your family suffers from schistosomiasis*, *does it impair your worth*? (EMIC2),
- *Would others think of a person with schistosomiasis being worthless/“being no human being”?* *(*EMIC4)
- *Would people in your village think that families with schistosomiasis patients are impaired in their worth*? (EMIC8)

According to the language expert conducting the backtranslation, the translation of *worth* in Kiswahili language “*thamani”* refers to an economical value, i.e. a price, which may also spark some misunderstanding.

Second, item EMIC5 “*Would knowing that someone has schistosomiasis have an adverse effect on others*?” was unclear to the participants as the “*adverse effect*” was not described in a concrete way. The “*adverse effect*” was translated into “*being dangerous*” according to the backtranslation.

Finally, a challenge in the translation of “possibly” could be overcome. According to a consulted Kiswahili language expert, *“inawezekana”* has the meaning of “*it is possible”* which may *always* be perceived to be true. This may lead to false-positive responses. Fortunately, this response option was ticked very seldom. Thus, the translation with “inawezekana” appears to be suitable. Yet, clearly presenting the response options in the beginning is recommended.

#### Operational equivalence

The interviewers who conducted the EMIC-CSS reported in the pilot study that they sometimes explained the items of the EMIC-CSS in more detail when the participants asked. To ensure a standardized procedure in the interviewing, it was agreed on having as little explanation on the items in the interview situation as possible.

Due to the numbers of illiterate participants (43.9% in pilot study, 27.5% in main study) the interview format proved to be a good approach. Further, it was very helpful that all interviewers spoke basic Kisukuma language as the majority of the participants in all study parts spoke Kisukuma language as mother tongue. Greetings in Kisukuma language allowed a welcoming atmosphere as reported by the interviewers. Still, interviewees not proficiently fluent in Kiswahili language were excluded from the assessment ensuring standardized interviews.

#### Measurement equivalence

- Floor and ceiling effects
- Reliability analysis: internal consistency
  ○ Cronbach’s alpha
  ○ item-total correlations
- Factor analysis
- Differences between the interviewers: Kruskal-Wallis test for independent samples

*Floor and ceiling effects*

Less than 15% of the data of the EMIC-CSS sum score were in the highest or lowest scores. Floor and ceiling effects (i.e., many participants responding in the lowest (floor) or highest (ceiling) numbers of scale) may limit content validity and reliability. If floor and ceiling effects occur, participants within highest or within lowest scores cannot be distinguished from each other. According to Terwee et al., if less than 15% score highest or lowest in a sample size of at least 50 participants, one can assume sufficient variability in the scale [32].

*Reliability analysis: Internal consistency and item selectivity*

The internal consistency of the EMIC-CSS scale was satisfying with a Cronbach’s alpha of α = 0.86 [43]. Item-total correlations were calculated to assess item selectivity of each item. Good item selectivity is described in literature to be .40 ≤ r ≤ .70 [44] and indicates that the item is effective in measuring stigma. Two items showed selectivity below .40: EMIC15 with *r* = .26 and EMIC1 with *r =* .32. Because Cronbach’s alpha would only increase from .857 to .861 or to .856, respectively if the items EMIC15 and EMIC1 were excluded, the low item selectivity of EMIC15 and EMIC1 could be neglected. Item-total-statistics can be seen in Table 6.

**Table 6:** Item-total-statistics.

Item	Scale mean if item deleted	Scale variance if item deleted	Corrected item-total-correlation	Squared multiple correlation	Cronbach’s alpha if item deleted
EMIC_1_	7.10	46.45	.33	**.22**	.856
EMIC_2_	6.93	44.16	.45	**.42**	.850
EMIC_3_	6.61	43.11	.49	**.46**	.849
EMIC_4_	7.17	44.92	.51	**.33**	.848
EMIC_5_	6.74	42.94	.51	**.41**	.848
EMIC_6_	7.26	44.92	.56	**.58**	.846
EMIC_7_	7.30	45.95	.49	**.61**	.849
EMIC_8_	7.21	44.60	.56	**.51**	.845
EMIC_9_	6.59	43.77	.43	**.37**	.853
EMIC_10_	7.06	43.70	.55	**.51**	.845
EMIC_11_	7.09	43.06	.63	**.67**	.841
EMIC_12_	6.95	42.40	.62	**.54**	.841
EMIC_13_	7.13	43.20	.63	**.65**	.841
EMIC_14_	7.27	45.38	.52	**.49**	.847
EMIC_15_	6.95	46.42	.26	**.15**	.861

If all items are included, the mean sum score is M = 8.35 (*SD* = 6.63).

*Factor analysis*

The principal component analysis with varimax rotation identified four factors according to the Kaiser-criterion, while the Scree test favors a one-factor solution [45,46]. This pattern is due to the fact that a first factor explains 35.5% of the variance, while factors 2 to 4 merely explain between 11.2% and 7.4% of the variance. While the four factors could in principle reflect different subscales (e.g. distinct types of stigma), their interpretation based on the content of the items does not yield clearly distinct concepts. The contents of the items in the third and fourth factor seem to be rather heterogeneous and therefore do not confirm the four-factor-structure (Table 7). In addition, the small number of items per factor renders it difficult to formulate clear subscales. Finally, order effects can be seen in the distribution of the items on the four factors combining those items to a factor which are assessed after each other, e.g. item 3, 4 and item 5. Accordingly, we support the one-factor-solution suggested by previous literature [27].

**Table 7:** Rotated component matrix.

Item	Component			
	1	2	3	4
13. Would having schistosomiasis cause a problem for a relative of that person to get married?	**.831**	.218	.093	.113
11. Would schistosomiasis be a problem for a person to get married?	**.812**	.213	.154	.049
12. Would schistosomiasis cause problems in an on-going marriage?	**.751**	.077	.250	.180
10. Would a family have concern about disclosure if one of their members had schistosomiasis?	**.748**	.084	.157	.069
14. Would having schistosomiasis cause difficulty for a person to find work?	**.706**	.269	.012	.077
7. Would others refuse to visit the home of a person affected by schistosomiasis?	.162	**.860**	.152	-.021
6.Would other people in your community avoid a person affected by schistosomiasis?	.249	**.799**	.118	.157
8. Would people in your village think that families with schistosomiasis patients are impaired in their worth?	.275	**.758**	.137	.146
9. Would schistosomiasis cause problems for the family?	.266	.119	**.771**	-.272
3. In your community, does schistosomiasis cause shame or embarrassment?	.074	.043	**.747**	.369
5. Would knowing that someone has schistosomiasis have an adverse effect on others?	.136	.223	**.660**	.188
4. Would others think of a person with schistosomiasis being worthless?	.166	.334	**.433**	.320
1. Would a person with schistosomiasis keep others from knowing, if possible?	.149	.036	.023	**.801**
2. If anyone in your family suffers from schistosomiasis, does it impair your worth?	.161	-.020	.436	**.644**
15. Would people buy food from a person affected by schistosomiasis?	.037	.262	.058	**.426**

*Differences between the mean scores of the interviewers*

The Kruskal-Wallis-test demonstrated a statistically significant overall difference in the EMIC-sum score between the different interviewers: χ2(3) = 32.129, *p* < .0001. The significant difference appeared between the second male interviewer and the other interviewers, as revealed by post-hoc Dunn Bonferroni tests. There were no significant differences between the other three interviewers. The second male interviewer shows a higher mean rank EMIC-sum score of 118.25, whereas the second female interviewer has 85.92 as mean rank EMIC-sum score, the first female interviewer 74.61 and the first male interviewer 66.00. The effect size for this difference ranges from moderate effect r = .31 between the second male interviewer and the first female interviewer up to a large effect between the second male interviewer and the first male interviewer r = .52. As there were no indications as to why sum scores were higher for the second male interviewer—e.g., whether he committed mistakes during the procedure or whether he happened to talk to people with higher overall scores—the data of the second interviewer were not excluded.

## Discussion

The aim of the present study was to validate the suitability of the adapted 15-item version of the EMIC-CSS to assess stigma towards schistosomiasis in the Mwanza region, Tanzania, using the cultural framework of Herdman and Fox-Rushby as reference [31].

When considering conceptual and item equivalence, all forms of stigma addressed in the EMIC-CSS *(concealment*, *shame*, *avoidance*, *process of discrediting*, *problems with getting married or ongoing marriage*, *problems with work*, *problems for family or other people*) were referred to and described repeatedly by the participants in the focus group discussions. These qualitative results were supported by quantitative results: 43% of the participants reported stigma. Hence, perceptions and attitudes of stigma towards schistosomiasis are existent under the survey participants [25]. A mean EMIC-CSS sum score of 8.35 underlines the notion that stigma related to schistosomiasis is a relevant construct in Mwanza region, Tanzania. However, the FGDs did not provide an equally clear picture for all aspects of stigma. For instance, while shame and embarrassment were mentioned in relation to schistosomiasis, it was also described by some as a normal disease. Additional research is therefore needed to address how these individual aspects contribute to stigma towards schistosomiasis. The aspects *problems with getting married or ongoing marriage* were perceived to be major topics for the participants of the FGDs. This is consistent with the scale assessing marriage problems with three different items. Further, *problems with work* were reported as major problem when suffering from schistosomiasis. However, it was not entirely clear whether work-related problems were due to schistosomiasis itself or to poor living conditions. The intersection of multiple stigmatizing conditions, especially that of stigma related to health conditions and stigma related to poverty, is an often-reported phenomenon [8,14,47] and thus, it does not question conceptional equivalence.

The concept of stigma itself deserves a closer look in the framework of the Tanzanian culture. Stigma in Kiswahili is *“unyanyapaa*” and reflects a well-known concept. The use of the word and concept *“unyanyapaa”* is especially prevalent in the context of HIV/AIDS and other highly stigmatized conditions. When looking at research on *“unyanyapaa”*, it is apparent that most studies focused on stigma towards HIV/AIDS and homosexuality, especially between men, also considering sexually transmitted diseases such as HIV/AIDS [48,49]. This inspires the question whether *“unyanyapaa”* is associated with severe forms of stigma. In the EMIC-CSS, the term “*unyanyapaa*” was not used, but it was referred to in explanations by the facilitators and participants in the FGDs. The associations with extreme forms of stigma would explain some negations of stigma and discrimination in the FGDs. Moreover, there is apparently no word for the concept of stigma in Kisukuma language which may contribute to a decreased understanding of the concept of stigma.

In general, our data support conceptual equivalence as well as item equivalence.

Semantic equivalence was confirmed in large parts in the expert discussions evaluating the interview process and in the backtranslation by a language expert. However, the expression “*thinking less of oneself”* referred to in three items (EMIC2, EMIC4, EMIC8) does not seem to have equivalent terms in Kiswahili culture. In the evaluation of the translation after the assessment, the items could be compared with the Kiswahili version of the EMIC-CSS used for albinism by De Groot and colleagues [34,35]. De Groot faced the same challenge and translated the term in EMIC2 different from the items EMIC4 and EMIC8. The term *“thamani”* used in this study in EMIC2, EMIC4 and EMIC8 was also selected by De Groot in EMIC2 in a broad assessment with several runs. Though the term is often referred to in an economic sense and thus may be misunderstood, the items EMIC2, EMIC4, EMIC8 did not show larger standard deviations than other items, suggesting that respondents did not understand these questions in very different ways. Hence, it may be well understood by the participants despite the connotation. The verb “*kudharau*” (“*to despise sb*.*”/”to ignore sb*.*”)* which is strongly associated with discrimination due to racism and used by De Groot and colleagues [34,50] for EMIC4 and EMIC8, or *“kunyanyapaa”* which is used in the context of HIV/AIDS-related stigma, was not investigated in this study.

The translation of the term “*adverse effect*” in EMIC5 was also challenging. The backtranslation showed that it was understood as being “*dangerous*”. In the Kiswahili EMIC-CSS for albinism by De Groot and colleagues, this item was removed entirely as it is related to the aspect of concealment which is not possible for people with albinism [34]. As the term is also relatively vague in English, this may be an item which leaves much room for interpretation. Yet, the translation into “*athari mbaya*” seems to be suitable, as it meets this broad meaning of an “*influence/impact/effect*” which is “*bad”* or even “*evil”*.

The response scale brought up questions on the translation of “*possibly”* into *“inawezekana”* and the translation of *“no”* into “*hapana*”. “*Inawezekana*” was selected as it is closest to the word “*possibly”*, though our language expert remarked that the response “*it is possible*” may be confirmed for almost any question. Yet, it was seldomly selected in this study and thus can be maintained in the response scale. “*No”* was translated with “*hapana”* which stands for a very strong negation with the connotation “*never”/” under no circumstances*”. A translation with *“hamna” (“no/there is nothing”)* could be a less strong negation which is used more often in everyday communication. To have a clear distance between the responses, “*hapana”* was used and is further recommended.

When considering operational equivalence in the EMIC-CSS implementation, particular aspects which have been discussed in previous studies were addressed: the interview format and its challenges and the response scale [27,34,35]. In order to include illiterate participants, the assessment with the EMIC-CSS was conducted via interviews, following the example of the EMIC-CSS used for stigma towards leprosy [27]. Interviews instead of self-administered questionnaires bear the risk of influence due to the social desirability bias. The social desirability bias describes the tendency of the interviewee to respond in a way they assume to be desired by the interviewer or society [51]. During FGDs, the facilitators were perceived to be experts on schistosomiasis, as reflected, for instance, in the participants’ requests for educational training etc. In case the participants considered the interviewers to be experts, this could further increase the chances of introducing social desirability bias, while for instance the sex, race and ethnicity and language use of the interviewers could introduce interview bias [52,53]. The measured effect of interviewers will be discussed when reflecting measurement equivalence.

A response scale with four options was chosen. First, this ensures comparability with previous research, as it has been the common response scale for the EMIC-CSS [10]. In addition, memorizing four response options seems ideal in a verbal interview situation in which the options cannot be re-read by participants. Yet, the response scale with the four options *(“Yes”*, *“Possibly”*, *“No”*, *“I don’t know”)* bears two risks: On the one hand, the low number of response options may lead to extreme responses. On the other hand, the responses “*Possibly”* and “*I don’t know”* may lead to indecisive response behavior. De Groot et al. used a five-point-scale for the assessment of stigma related to albinism and specifically asked about the frequency of the behaviors or experiences described in the items (i.e., it rarely/sometimes/often happens) [34]. The added value of these responses by De Groot is that they may represent the gray area of the phenomenon in society better. This may make the questions easier to answer.

The closed response format of the EMIC-CSS shows to be very useful for controlling bias due to language barriers, e.g. a different mother tongue. Research suggests that sensitive subjects like emotions can be expressed most openly in mother tongue [54,55]. Since in Mwanza region many communities rather speak Kisukuma language as mother tongue [56], this may have influenced results of the FGDs with participants being hesitant to disclose themselves or connotations of statements being lost.

The validation has shown sufficient psychometric properties ensuring measurement equivalence. Internal consistency of the adapted stigma scale as a whole was confirmed and no floor and ceiling effects were found. In contrast to some previous findings, our results suggest the existence of four subscales when following the Kaiser-criterion [46]. The four factors of the EMIC-CSS discovered in this study may reflect different aspects of stigma related to schistosomiasis. These are *problems in marriage*, *family and work* (EMIC10,11,12,13,14), *social disclosure* (EMIC6,7,8), *shame and process of discrediting* (EMIC3,4,5,9) and *concealment and social distance* (EMIC1,2,15). As the first factor explains 35.5% of the variance and the item number for second, third and fourth factor is too small for independent subscales, the one-dimensionality is presumed. Further research on the factor-structure of the EMIC-CSS is recommended.

Since the interviewer bias was explicitly considered in the present investigation, a highly standardized procedure in the interview situations was discussed in briefings and established in the interview situations. To avoid race and ethnicity interviewer effects, White persons were excluded from interview situations. Davis and colleagues [53] already stated the importance of measuring and controlling interviewer bias in public health surveys, e.g. race and ethnicity effects [53]. For instance, a White person interviewing a Black respondent may lead to a higher social desirability bias and to reduced openness [53]. Yet, there is little research on the question whether matching of interviewer and interviewee would improve data quality [53].

### Limitations and recommendations

In the present study, construct validity was not assessed due to the lack of a second stigma assessment tool together with the EMIC-CSS. To keep participants motivated and concentrated during the interview, interviewers were advised to keep the interview short, which is why we decided against a second scale. Similarly, we did not investigate retest-reliability, as retesting would have gone beyond the scope of this research project. One interviewer in the present study showed significantly different results in the interviews compared to the others. The influence of the second male interviewer cannot be sufficiently explained but there was no indication for an exclusion of his data. Another limitation is the convenience sampling on the data collection days of the pilot and main implementation of the EMIC-CSS. Convenience sampling bears the risk of selection bias, e.g. having those people more likely participate who are more affected by the topic, impairing generalizability. Yet, across the whole data collection, the size of each community sample was kept mostly equal, in order to allow for higher generalizability in Mwanza region.

One limitation of this study is that the backtranslation took place after the main data assessment due to technical oversight. The backtranslation being conducted after the data collection meant that several insights gained from the backtranslation could not be considered in the main data collection. Accordingly, some alternative translations suggested in the course of backtranslation could not be tested in the main analysis, e.g. a different translation for *adverse effect* or *worth*.

Despite the above-mentioned limitations, the findings of this study allow several recommendations to be made: First, paying closer attention at the items EMIC2, EMIC4 and EMIC8 containing the term *thinking less of sb* is recommended. A pilot run could serve to compare the following three versions of the EMIC-CSS: one using this translation (*thamani*), one using *kudharau* for the translation of *thinking less of sb*. and one using *kunyanyapaa*.

We recommend good interviewer training and emphasis on establishing standardized settings when applying the EMIC-CSS.

An often-discussed aspect of the EMIC-CSS is the response scale. This validation study showed sufficient variance in the three-point-response scale (“yes”, “possibly”, “no”) with the additional option “I don’t know”. Additionally, the use of visual support in the interview situation e.g., showing three different symbols, is advisable.

Though social desirability and the interviewer bias may influence stigma assessment in interviewer settings, the conduction of interviews for schistosomiasis-related stigma assessment is recommended. As schistosomiasis is a poverty-related disease and poverty is associated with illiteracy [1,57,58], it is important to include the most vulnerable individuals for schistosomiasis. The present study took particular care in this regard as the interviews took place in twelve different communities in the lakeshore areas which are particularly remote and dependent on lake water use due to lack of safe alternative water sources; hence there is a high vulnerability towards contracting schistosomiasis disease. This validation study bears a high generalizability for people at risk of schistosomiasis in Tanzanian culture because it addressed representative vulnerable communities.

## Conclusion

The present results suggest cultural equivalence of the EMIC-CSS and thus introduces a valid instrument for public stigma related to schistosomiasis. Future studies should additionally address the particular roles of specific aspects, e.g. the burden for the family, or even different forms of schistosomiasis in the development of stigmatization, e.g. female genital schistosomiasis. The use of additional questionnaires that assess participants’ knowledge on schistosomiasis and its symptoms in order to get a clearer idea on the preconditions of stigmatization is recommended. To achieve a full picture of stigma related to schistosomiasis, experienced and internalized stigma should be addressed via self-stigma scales in future research.

The use of the EMIC-CSS has been proven to be useful in the context of stigma assessment towards schistosomiasis in Mwanza region. The scale was validated and the results confirm the existence of stigma towards schistosomiasis.

## Supporting information

S1 Fig Cultural adaption and validation process. (TIF)

S1 Table Differences between the interviewers. (XLSX)

S1 Text Interview material Swahili and English. (DOCX)

S2 Text Guidelines of the focus group discussions Swahili and English. (DOCX)
