# Supplementary figures and images for: Cultural adaption and validation of the Explanatory Model Interview Catalogue–Community Stigma Scale in the assessment of public stigma related to schistosomiasis in lakeshore areas of Mwanza region, Tanzania
Source: PLoS Negl Trop Dis. 2023 Aug 14;17(8):e0011534. doi: 10.1371/journal.pntd.0011534 (PMC10449129; doi:10.1371/journal.pntd.0011534)

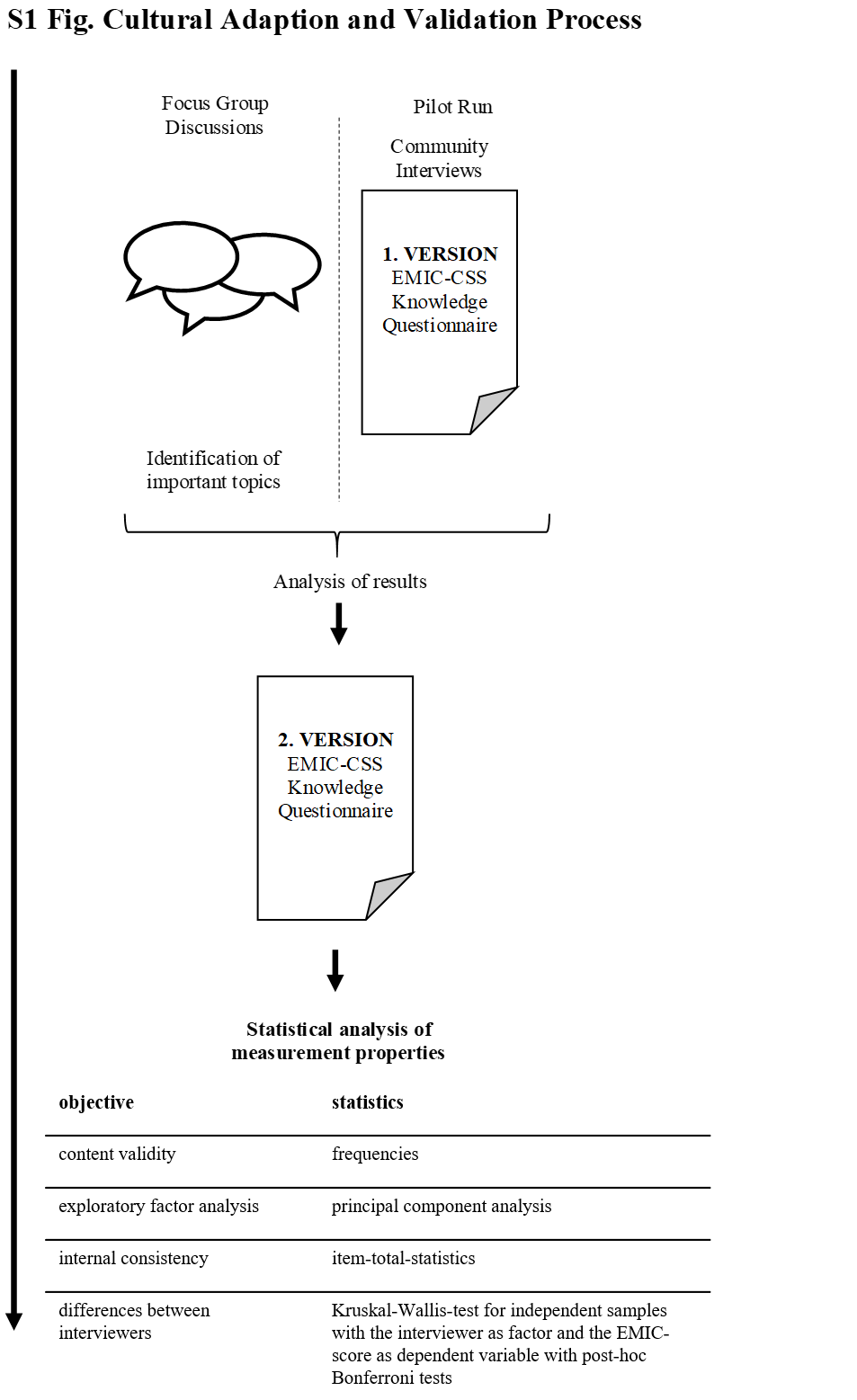

Supplement: S1 Fig — (TIF) [file pntd.0011534.s001.tif]
