# Supplementary material for: Cultural adaption and validation of the Explanatory Model Interview Catalogue–Community Stigma Scale in the assessment of public stigma related to schistosomiasis in lakeshore areas of Mwanza region, Tanzania
Source: PLoS Negl Trop Dis. 2023 Aug 14;17(8):e0011534. doi: 10.1371/journal.pntd.0011534 (PMC10449129; doi:10.1371/journal.pntd.0011534)
Supplement: S1 Text — (DOCX) [file pntd.0011534.s003.docx]

**S1 Text. Interview Material Swahili and English**

**Dodoso la Utafiti kuhusiana na unyanyapaa kwa wagonjwa wa kichocho katika jamii**

**Utangulizi**

Kwa sasa tunaendesha utafiti ambao tunataka kujifunza zaidi kuhusu athari za kichocho katika maisha ya kila siku katika mkoa wa Mwanza. Tunataka kutathmini ufahamu na hisia zako kuhusiana na kichocho. Mwanzoni tutauliza maswali kuhusu ushirikiano wa kijamii wa wagonjwa wa kichocho katika jamii. Baadaye tutauliza maswali kuhusu uelewa wako wa ugonjwa wa kichocho na hisia zako kuhusu ugonjwa wa kichocho ikilinganishwa na magonjwa mengine yaliyokatika maeneo haya. Tunavutiwa na maoni yako na uzoefu wako, kwa hiyo hakuna majibu yasiyo sahihi

Taarifa zote unazozitoa zitabaki siri na hazitahusishwa na jina lako. Kushiriki katika mahojiano haya ni kwa hiari. Ikiwa kwa sababu yoyote hujisikii vizuri, mahojiano yanaweza kusitishwa, na maelezo yako yataondolewa.

Una maswali yoyote kuhusu mjadala huu? Ikiwa una maswali yoyote kuhusu haki zako katika utafiti au suala lingine lolote, unaweza kuwasiliana na Dkt. Humphrey Mazigo.. Ushiriki wako katika utafiti huu utachukua muda wa dakika 30. Tafadhali tunaomba uweke sahihi yako hapa chini kuonyesha kwamba umeelewa haki yako juu ya kushiriki katika utafiti huu na umeridhia kushiriki kwa hiari yako.

**………………………………………. …………………………………………….
Sahihi yako Tarehe na mahali**

Introduction

We are currently running a study in which we want to learn more about the impact of schistosomiasis in daily life in Mwanza region. We want to assess the understanding and feelings towards schistosomiasis. In the beginning we will ask questions concerning social interactions of schistosomiasis patients in the community. Afterwards we will ask some questions about your understanding of schistosomiasis and your feeling towards schistosomiasis compared to other diseases. We are interested in your personal opinion and experiences, so there are no wrong answers.
All information you provide will remain confidential and will not be associated with your name.  Participation in this interview is voluntary. If for any reason you do not feel comfortable, the interview may be stopped, and your information will be discarded.

Do you have any questions about this discussion? If you have any questions about your rights in the study or any other issue, you may contact Humphrey Mazigo.

Your participation in this study will take approximately 30 minutes. Please indicate with your signature on the space below that you understand your rights and agree to participate.

**………………………………………. …………………………………………….
Your signature Date and place**

| **Taarifa za binafsi za kijamii za mshiriki (Sociodemographics)** | | | |
| --- | --- | --- | --- |
| 1. Jinsia ya mshiriki (Sex of Respondent) | Mke (Female)………………………………..0  Mume (Male)…………………………………1 | |  |
| 1. Umri wa mshiriki (mwaka ulikamilika)   [Age (in completed years)] |  | |  |
| 1. Kiwango cha elimu (Level of education) | Hakuwahi kuhudhuria shule au kuhitimu elimu ya msingi………………1  [*Illiterate (didn’t attend school or completed primary school*…............1]  Amehitimu elimu ya msingi…………..2  [*Completed primary school education………………………………………2*]  Hakuhitimu elimu ya sekondari……..3  [*Not completed Secondary education………………………………………3*]  Amehitimu elimu ya sekondari……….4  [*Completed secondary education………………………………………4*]  Amehitimu elimu ya chuo………………5  [*Completed college……………………….5*]  Amehitimu elimu ya Chuo Kikuu……6  [*Education of higher learning*  *Institutions……………………………………6*] | |  |
| 1. Hali ya mahusiano ya ndoa (Marital status) | Hana mahusiano ya ndoa………………1  [*Single…………………………………………..1*]  Ameoa/ameolewa…………………………2  [*Married………………………………….…….2*]  Wanaishi pamoja bila kufunga ndoa………………………………………………3  [*Consensual union………………………...3*]  Wametalakiana...…………………………..4  [*Divorced………………………………………4*]  Wameachana………………………………..5  [*Separated……………….......................5*]  Mjane/Mgane……………………………….6  [*Widow/widowed…………………….……6*] | |  |
| 1. a. Kaya yako ina idadi ya watu wagapi  [*Number of people living in household*]   b. Idadi ya watu wazima wanaoishi kwenye kaya hii [*Number of adults living in household*]  c. Idadi ya watoto wanaoishi kwenye kaya hii [*Number of children living in household*] | 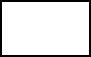a. Idadi ya watu kwenye kaya  b. Idadi ya watu wazima  [*Number of adults*]  c. Idadi ya watoto  [*Number of children*] | |  |
| 1. Kazi (Occupation) | Mkulima…………………………………………1  [*Peasant………………………………………..1*]  Mvuvi/mchakata samaki………………..2  [*Fishers/fish processors…………………2*]  Mama wa nyumbani………………………3  [*Housewife…………………………...........3*]  Mtumishi wa umma……………………….4  [*Public employee…………………………..4*]  Mtumishi wa sekta binafsi…………….5  [*Private sector employee……………....5*]  Mfanyabiashara…………………………….6  [*Businessman/woman………………....6*]  Mfanyabiashara ndogo ndogo……….7  [*Petty business……………………………...7*]  Nyinginezo………………………………….-99  [*Others……….………………………..……-99*]  Zitaje:………………………………………………  [Specify……………………………………..……] | |  |
| 1. Kabila la mshiriki (Ethnic group of respondent) | Msukuma………………………………………1  [*Sukuma…………………………….………….1*]  Mkara……………………………………………2  [*Kara…………………………………………….2*]  Mkerewe……………………………………….3  [*Kerewe…………………........................3*]  Mzinza……………………………………………4  [*Zinza……………….……………………………4*]  Mhaya……………………………………………5  [*Haya………….…………………………………5*]  Mjita………………………………………………6  [*Jita..................................................6*]  Nyingine……………………………………-99  [*Other………………………..................-99*]  Taja:……………………………………………….  [Specify…………………………………………] | |  |
| 1. Dhehebu la mshiriki [Religion of respondent] | Mkatoliki……………………………………….1  [*Catholic………………………………………1*]  Mporestanti………………………………….2  [*Protestant…………………………………..2*]  Muislamu………………………………………3  [*Muslim………………………………………..3*]  Msabato………………………………………..4  [SDA……………………………………………..4]  Mpentekoste…………………………………5  [*Pentecostal………………….................5*]  Hana dini/mpagani…………………………6  [*Pagan………………………………………….6*]  Nyingine……………………………………-99  [Other………………………………………-99]  Taja:……………………………………………….  [Specify:…………………………………………] | |  |
| **Utangulizi: Maswali kuhusu ugonjwa wa kichocho (kisambale)** | | | |
| 1. Je, unafahamu watu walio na maambukiizi ya ugonjwa wa kichocho katika jamii yenu/Kijiji/mtaani kwenu?  [*Do you know people out of your community having schistosomiasis?*] | Ndiyo  (Yes) | Hapana  (No) | Sijui  (Do not know) |
| 1. Ni dalili gani/zipi mgonjwa/wagonjwa wa kichocho wanazo/wanaonyesha?  [*Which symptoms do schistosomiasis patient(s) present?*]   Weka alama ya vyema kwenye dalili iliyotajwa *(tick the symptoms which are mentioned)* | Maumivu ya tumbo……………….……...1  [*Abdominal pains/colic………………….1]*  Kuharisha/kupata haja kubwa laini..2  [*Diarrhoea/loose stool..……...…..……2*]  Damu kwenye haja kubwa……………..3  [*Blood in stool……............................3*]  Kutapika damu………………………………4  [Vomiting blood………………..………….4]  Kuvimba tumbo/tumbo kujaa maji..5  [*Swollen abdomen/Ascites..............*5]  Kukojoa damu……………………………….6  [*Urinating blood……………………………6]*  Maumivu wakati wa kukojoa………..7  [*Painful micturition……….................7*]  Dalili zinginezo…………………………….99  Others…………………………………………99] | |  |
| 1. Umesikia mara ngapi kuhusu watu wenye mojawapo ya dalili hizo katika jamii yako?  *[How often have you heard of people of your community showing one of these symptoms?]* | Mara kwa mara  [Always/ often] | Mara chache (Sometimes) | Nadra  [Rarely/ once] |

| Maswali (QUESTIONS) | Kiwango cha majibu (Response Scale) | | | |
| --- | --- | --- | --- | --- |
| **Maswali ya kupima unyanyapa dhidi ya ugonjwa wa kichocho katika jamii [Explanatory Model Interview Catalogue – Community Stigma Scale^[[1]](#footnote-1)^]** | Ndiyo  [Yes]  (2) | Inawezekana  [Possibly] (1) | Hapana  [No]  (0) | Sijui  [Do not know]  (-99) |
| 1. Je, mtu mwenye maambukizi ya ugonjwa wa kichocho atawaficha wengine kujua kama anaugua ugonjwa wa kichocho?  [*Would a person with schistosomiasis keep others from knowing, if possible*?] |  |  |  |  |
| 1. Kama mmoja wa wanafamilia yako atakuwa anaugua ugonjwa wa kichocho, je utajisikia kupungukiwa thamani yako?  [*If a member of your family had schistosomiasis, would you think less of yourself*?] |  |  |  |  |
| 1. Katika jamii/Kijiji/mtaa wenu, je ugonjwa wa kichocho husababisha aibu au usumbufu? [ *In your community, does schistosomiasis cause shame or embarrassment*?] |  |  |  |  |
| 1. Je, watu wengine wanawachulia watu wanaougua ugonjwa wa kichocho hawana utu/thamani?  [*Would others think less of a person with schistosomiasis*?] |  |  |  |  |
|  | Ndiyo  [Yes]  (2) | Inawezekana  [Possibly] (1) | Hapana  [No]  (0) | Sijui  [Do not know]  (-99) |
| 1. Je! Kujua kwamba mtu ana ugonjwa wa kichocho ina athari mbaya kwa wengine  [*Would knowing that someone has schistosomiasis have an adverse effect on others*?] |  |  |  |  |
| 1. Je, Watu wengine katika Kijiji chenu wanaweza kumtenga/kumnyanyapaa mtu aliyeathiriwa na ugonjwa wa kichocho  [*Would other people in your community avoid a person affected by schistosomiasis*?] |  |  |  |  |
| 1. Je, watu wanaweza kukataa kutembelea nyumba/makazi ya mtu anayeugua ugonjwa wa kichocho?  [*Would others refuse to visit the home of a person affected by schistosomiasis*?] |  |  |  |  |
| 1. Je, watu katika jamii/kijiji chenu wanaziona familia zenye mgonjwa/wagonjwa wa kichocho hazina utu/thamani?  [*Would people in your community think less of the family of a person(s) with schistosomiasis*?] |  |  |  |  |
| 1. Je, ugonjwa wa kichocho unaweza kusababisha matatizo katika familia?  [*Would schistosomiasis cause problems for the family*?] |  |  |  |  |
| 1. Je, familia inaweza kuwa na wasiwasi kuhusu kutoa taarifa kwa uwazi kama moja wa wanafamilia anaugua ugonjwa wa kichocho?  [*Would a family have concern about disclosure if one of their members had schistosomiasis*?] |  |  |  |  |
| 1. Je, ugonjwa wa kichocho unaweza kuwa tatizo/kikwazo kwa mtu kuoa/kuolewa?  [*Would schistosomiasis be a problem for a person to get married*?] |  |  |  |  |
| 1. Je, ugonjwa wa kichocho unaweza kusababisha matatizo katika ndoa inayoendelea?  [*Would schistosomiasis cause problems in an on-going marriage*?] |  |  |  |  |
| 1. Je, kuwa na kichocho kunaweza kusababisha matatizo kwa ndugu wa mtu kuolewa/kuoa?  [*Would having schistosomiasis cause a problem for a relative of that person to get married*?] |  |  |  |  |
| 1. Je, kuwa na kichocho kunaweza kuwa kikwazo kwa mtu kupata ajira/kazi?  [*Would having schistosomiasis cause difficulty for a person to find work*?] |  |  |  |  |
| 1. Je, watu wanaweza kununua chakula toka kwa mtu anayeugua ugonjwa wa kichocho?  [*Would people buy food from a person affected by schistosomiasis*?] |  |  |  |  |

| Maswali [QUESTIONS] | Kiwango cha majibu [Response Scale] | | | |
| --- | --- | --- | --- | --- |
| 1. **Kuangalia machoni [Eye Contact]** | Ndiyo  [Yes]  (2) | Inawezekana  [Possibly] (1) | Hapana  [No]  (0) | Sijui  [Do not know]  (-99) |
| 1a. Je, watu wengine watakwepa kumwangalia usoni mtu anayeugua ugonjwa wa kichocho?  [*Would other people avoid eye contact with a person affected by schistosomiasis*?] |  |  |  |  |
| 1b. Je watu watamwangalia kwa mshangao mtu anayeugua ugonjwa wa kichocho?  [*Would other people stare at a person affected by schistosomiasis*?] |  |  |  |  |

| Maswali [QUESTIONS] | Kiwango cha majibu [Response Scale] | | | |
| --- | --- | --- | --- | --- |
| 1. **Mahitaji ya msingi ya kijamii [Basic Social Need “Control”]** | Ndiyo  [Yes]  (2) | Inawezekana  [Possibly] (1) | Hapana  [No]  (0) | Sijui  [Do not know]  (-99) |
| 2a. Je, ugonjwa wa kichocho unaweza kupunguza kiwango cha mtu kuchangia katika maendeleo ya jamii?  [*Would schistosomiasis reduce the extent to which a person can contribute to community life*?] |  |  |  |  |
| 2b. Je, ugonjwa wa kichocho unaweza kupunguza kiwango cha mtu kuchangia katika maendeleo ya familia yake?  [*Would schistosomiasis reduce the extent to which a person can contribute to the well-being of his family*?] |  |  |  |  |
| 2c. Je, ugonjwa wa kichocho unaweza kumfanya mtu akajiona hana uwezo, (Kwa mfano-kufanya kazi, kujenga familia, kuchangia maendeleo ya jamii)?  [*Would schistosomiasis make a person feel powerless*?] |  |  |  |  |

| Maswali ya wazi [OPEN QUESTIONS ] |
| --- |
| 1. **Ufahamu kuhusu ugonjwa wa kichocho [Illness Explanatory Model*]** |
| 3a. Je, watu wanaambukizwaje kichocho? [*How do people become infected*?] |
| 3b. Kwanini unafikiri watu hupata ugonjwa wa kichocho? [*Why do you think people get schistosomiasis*?] |
| 3c. Kwa nini watu wengine wanafikiri watu hupata kichocho? [Why do other people think people get schistosomiasis?] |

| Mswali ya wazi (OPEN QUESTIONS) |
| --- |
| 1. **Jinsia [Gender]** |
| 4a. Kwanini unafikiri wanaume wanapata kichocho? [Why do you think men get schistosomiasis?] |
| 4b. Kwanini unafikiri wanawake wanapata kichocho? [*Why do you think women get schistosomiasis*?] |
| 4c. Kwanini wengine wanafikiri wanaume wanapata kichocho? [*Why do others think men get schistosomiasis*?] |
| 4d. Kwa nini wengine wanafikiri wanawake wanapata kichocho*? [Why do others think women get schistosomiasis?]* |

| Maswali ya wazi [QUESTIONS] | Kiwango cha Majibu (Response Scale) | | | |
| --- | --- | --- | --- | --- |
| 1. **Afya ya uzazi**   **[Reproductive health / Sexual Life]** | Ndiyo  [Yes]  (2) | Inawezekana  [Possibly] (1) | Hapana  [No]  (0) | Sijui  [Do not know]  (-99) |
| 5a. Je, unafikiri ugonjwa wa kichocho ni miongoni mwa magonjwa yanayoambukizwa kwa njia ya kujamiana/ ngono?  [*Do you think schistosomiasis is among sexual transmitted diseases?]* |  |  |  |  |
| 5b.   1. Je, unafikiri ugonjwa wa kichocho huathiri uwezo wa kujamiana / kufanya tendo la ndoa kwa mtu anayeugua ugonjwa huo?   [*Do you think schistosomiasis affects the sexual performance of people having schistosomiasis*?] |  |  |  |  |
| 5b.   1. Kama jibu ni ndiyo kwa swali 5b, ni kwa njia zipi? [*If the answer of question 5b is yes, in which way?]* | Kupungua kwa hamu ya kufanya tendo la ndoa………...1  [*Reduced sexual desire……….1]*  Kupungua kwa nguvu za kiume …………………………………………….2  [(reduced) *Manhood……………2*]  Maumivu wakati wa kujamiana kwa wanaume……………………..3  [pain during sexual intercourse for men…………………………….….3]  Maumivu wakati wa kujamiana kwa wanawake…………………..…4  [pain during sexual intercourse for women……………………………4]  Nyingine……………………………-99  [other………………………………-99] Zitaje:……………………………………..  [Specify…………………………….……] | | |  |
| 5c.   1. Je, unafikiri ugonjwa wa kichocho huathiri via / viungo vya uzazi kwa watu wanaougua ugonjwa huo?  [*Do you think schistosomiasis affects the reproductive organs of people having schistosomiasis*?] |  |  |  |  |
| 5c.   1. Kama jibu ni ndiyo kwa swali 5c, ni kwa njia zipi? [*If the answer of 5c is yes, in which way?]* | Ugumba kwa wanawake……….1  [infertility of females *.............1*]  Ugumba kwa wanaume…………2  [infertility of males……………….2]  Nyingine……………………………-99  [other………………………………-99]  Zitaje:……………………………………  [Specify…………………………………] | | |  |

| Maswali ya wazi [QUESTIONS] | | | |
| --- | --- | --- | --- |
| 1. **Maelezo kuhusu kichocho [Particularities about Schistosomiasis]** | | | |
| 6a. Nini kinakufanya uwe na woga/hofu/wasiwasi kuhusu kichocho? [What makes you worry most about schistosomiasis?] | | | |
| 6b. Unafikiri kuwa na ugonjwa wa kichocho ni bora kuliko kuwa na ugonjwa wa malaria? [Do you think having schistosomiasis is better than having Malaria?] | Ndiyo  [Yes] (1) | Hapana  [No]  (0) | Sijui  [Do not know]  (-99) |
| 6b.   1. Kwanini [Why?] | | | |
| 6c.   1. i) Unafikiri kuwa na kichocho ni bora kuliko kuwa na ugonjwa wa Ukimwi? [Do you think having schistosomiasis is better than having HIV/Aids?] | Ndiyo  [Yes] (1) | Hapana  [No]  (0) | Sijui  [Do not know]  (-99) |
| 6c.   1. Kwanini [Why?] | | | |
| 6d.   1. Unafikiri kuwa na kichocho ni bora kuliko kuwa na minyoo ya tumbo/michango? [Do you think having schistosomiasis is better than having diseases Soil Transmitted Helminths?] | Ndiyo  [Yes] (1) | Hapana  [No]  (0) | Sijui  [Do not know]  (-99) |
| 6d.   1. Kwanini [Why?] | | | |

1. Peters RMH, Dadun, Van Brakel WH, Zweekhorst MBM, Damayanti R, Bunders JFG, et al. (2014) The Cultural Validation of Two Scales to Assess Social Stigma in Leprosy. PLoS Negl Trop Dis 8(11): e3274. https://doi.org/10.1371/journal.pntd.0003274 [↑](#footnote-ref-1)
