# Supplementary material for: Cultural adaption and validation of the Explanatory Model Interview Catalogue–Community Stigma Scale in the assessment of public stigma related to schistosomiasis in lakeshore areas of Mwanza region, Tanzania
Source: PLoS Negl Trop Dis. 2023 Aug 14;17(8):e0011534. doi: 10.1371/journal.pntd.0011534 (PMC10449129; doi:10.1371/journal.pntd.0011534)
Supplement: S2 Text — (DOCX) [file pntd.0011534.s004.docx]

**S2 Text. Guidelines of the Focus Group Discussions Swahili and English**

**Muongozo wa majadiliano ya kikundi**

**Utangulizi**

Habari za leo.

Naitwa..................................Natokea………………………….....Ningependa kujifunza kuhusu mawazo na hisia zenu juu ya ugonjwa wa kichocho.

Tunafanya kazi na GLRA(Taasisi inayoshughulika na ugonjwa wa ukoma na magonjwa yasiyo pewa kipaumbele) na ni sehemu ya mradi wa kudhibiti ugonjwa wa kichocho ambao unaendelea kutekelezwa katika wilaya ya Ilemela na Nyamagana kupitia GLRA.

Lengo la Mazungumzo haya ni kukusanya taarifa zitakazosaidia kuboresha mradi.Tunataka kujifunza kuhusu uelewa wenu juu ya ugonjwa wa Kichocho.

Tutazungumza nanyi kwa muda wa takribani saa moja na nusu (1:30). Tunaomba kurekodi majadiliano yetu nanyi kwa kutumia kinasa sauti, ili itusaidie kupata kwa ufasaha taarifa zote mtakazo tupatia. Taarifa zote mtakazo tupatia zitabaki kuwa siri na hazitohusishwa na majina yenu. Kwa ajili ya kutunza kumbukumbu tutawaomba kama ni sawa kupiga picha au la.Tafadhali tujulishe kama si sawa kwako.

Ushiriki katika mahojiano haya ni wa hiyari.Yeyote ambaye hayuko tayari kushiriki anaruhusiwa kuondoka, na endapo mtu yeyote atahitaji kutokuendelea na majadiliano wakati majadiliano yanaendelea tafadhali anijulishe.Washiriki wako huru kutoa maoni yao.Hakuna jibu lisilo sahihi.Usisite kuuliza swali lolote wakati wa mahojiano.Tafadhali tunaomba azungumze mshiriki mmoja baada ya mwingine.

Je, mna swali lolote kuhusu mahojiano haya? Endapo una swali lolote kuhusu haki zako juu ya utafiti huu au juu ya suala lingine unaweza kuwasiliana na Dr, Humphrey Mazigo.

1. **Mwanzo**

Tafadhali tuambie kwa kifupi kuhusiana na jamii yenu

- Ni zipi fursa za kiuchumi kwenye jamii yenu?
- Mavuno yamekuaje? Msimu wa mvua ulikua mzuri?
- Ni yapi matatizo makuu kwa sasa ambayo mnakumbana nayo katika jamii yenu?

1. **Mtazamo juu ya ugonjwa wa Kichocho kwa kulinganisha na magonjwa mengine**
   - Ni magonjwa yapi makuu kwasasa ambayo yanatokea mara kwa mara kwenye jamii yenu?
   - Tafadhali tuambie kuhusu ugonjwa wa Kichocho. Ni kwa kiwango/ukubwa gani mnauchukulia ugonjwa wa kichocho kama tatizo katika jamii yenu?
   - Ni kwa kiwango/ukubwa gani mnauchukulia ugonjwa wa kichocho kama tatizo katika jamii yenu?
2. Ukilinganisha na magonjwa mengine ( mfn. Malaria au Ukimwi)
3. **Mtazamo/Hisia juu ya Ugonjwa wa Kichocho**
    Tafadhali tuambie mawazo/ hisia zenu kuhusiana na ugonjwa wa Kichocho
   - Ukifikiria kuhusu ugonjwa wa Kichocho, ni mawazo gani/hisia gani mnapata?
     1. Kwanini?
     2. Ni kwa namna gani hisia/ mawazo hizo/hayo zinawaathiri katika maisha yenu ya kila siku?
     3. Mawazo yako na hofu zako yanahusiana moja kwa moja na ugonjwa au yanahusiana zaidi na hali ya maisha yanayohusiana na ugonjwa? (dodosa kuhusu mazingira ya kimaskini, kuhusu kutengwa au kunyanyaswa)
4. **Kutambua ugonjwa wa Kichocho/ Kuzungumzia ugonjwa wa kichocho**
   - Unamfahamu mtu yeyote katika mazingira yenu ambaye ana ugonjwa wa Kichocho?
   - Unawezaje kujua kama mtu ana Kichocho?
   - Ni nini mnaona kama sababu hatarishi zinazopelekea ugonjwa wa Kichocho?
     1. Kazi? Elimu?Makazi?Tabia?
   - Ni kwa namna gani watu wanazungumzia Kichocho?
   - Watu wanawazungumziaje wagonjwa wa kichocho? Mf. Mtu unaye mfahamu ana (amewahi kua na) kichocho?
     1. Unadhani kuna tofauti endapo mwanamke au mwanaume ameambukizwa ugonjwa wa kichocho? Tafadhali elezea.
5. **Kundi lililo hatarini zaidi**
   - Nani/ Kundi gani la watu katika jamii yenu unadhani wako katika hatari kubwa zaidi ya kuambukizwa ugonjwa wa Kichocho?
   - Unadhani ni **(Nani kati ya)** wanaume na wanawake walio katika hatari zaidi ya kupata ugonjwa wa kichocho?
     1. Tafadhali elezea kwanini?
   - Unadhani nani anaathirika Zaidi kutokana na ugonjwa wa kichocho kati ya wanaume au wanawake?
     1. Tafadhali elezea kwanini
6. **Hatua za Kukabiliana na ugonjwa wa Kichocho**
   - Kama utakuwa na dalili zinazohusiana na ugonjwa wa Kichocho, nani wa kwanza utamwamini na kumuomba ushauri? (dodosa Zaidi kuhusu kupata huduma za tiba,Hospitali vs waganga wa jadi)
     1. Kwanini?
     2. Unadhani kuna tofauti kati ya wanawake na wanaume katika kuweka wazi hali ya afya zao juu ya dalili zinazohusiana na ugonjwa wa Kichocho? Tafadhali elezea.
     3. Je mnazo dawa za kienyeji / asili mnazotumia kutibu dalili zinazohusiana na ugonjwa wa Kichocho? Tafadhali fafanua?
   - Kwanini unafikiri Kichocho kinaendelea kuwa tatizo kubwa katika jamii yenu?

Asanteni, Tumejadili mambo mengi yanayohusu ugonjwa wa kichocho katika jamii yenu, je, kuna kitu chochote kinachohusu ugonjwa wa kichocho ambacho ni muhimu na hatujakigusia katika majadiliano yetu?

Asanteni kwa muda wenu na michango yenu.

**Focus Group Discussion Guidelines (English)**

**Introduction:**

Good day. I am ….…..... I’m from …......and wish to learn about your thoughts and feelings towards schistosomiasis.
We are working for GLRA and are part of the Schistosomiasis Control Project, which is being implemented in your district through GLRA*.* The objective of this session is to collect information to help improve the project. We want to learn about your understanding of schistosomiasis.
We will talk to you for about 1,5 hours. We will record our discussion with you using a voice recorder to allow us to accurately capture the information you provide. All information you provide will remain confidential and will not be associated with your name. For documentation purpose we would like to ask whether it is ok for you to take pictures. Please let us know, if it is not ok for you.
Participation in this interview is voluntary. Whoever is not willing to participate may leave the session now or at any time during the discussion. Participants are free to express their opinions. There is no right or wrong answer. Do not hesitate to ask any question during the discussion. Please speak one person at a time.

Do you have any questions about this discussion? If you have any questions about your rights in the study or any other issue, you may contact Dr. Humphrey Mazigo.

1. **Getting started**

Please tell us a bit about your community

- How are the economic opportunities in your community?
- How has the harvest been? Was the rainy season good?
- What are the **current major problems** that you encounter in the community?

1. **Perception of Schistosomiasis compared to other diseases**
   - What are the **current major diseases** that often occur in the community?
   - Please tell us about the disease schistosomiasis. How serious do you perceive schistosomiasis as a problem in your community?
   - How serious do you perceive schistosomiasis as a problem in your community
     1. Compared to other diseases (e.g. malaria or HIV)
2. **Thoughts/Feelings about schistosomiasis**
   Please tell us about your thoughts/emotions about schistosomiasis
   - If you think of schistosomiasis, which thoughts/emotions do you have?
     1. Why?
     2. How do these thoughts affect you in your daily life?
     3. Are your thoughts and concerns directly related to the disease or rather to the living conditions associated with the disease?
3. **Recognizing Schistosomiasis/ Talking about schistosomiasis**
   - Do you know somebody in your surrounding who has schistosomiasis?
   - How can you tell who has schistosomiasis?
   - What do you see as typical risk factors for schistosomiasis?
     1. Work? Education? Housing? Behaviour?
   - How do people talk about schistosomiasis?
   - How do people talk about schistosomiasis patients, e.g. the person you know having (had) schistosomiasis?
     1. Would you think it makes a difference whether a man or a women is infected? Please explain.
4. **Vulnerability**
   - Who/which group of people in your communities do you think are at particular risk to be or become infected with schistosomiasis?
   - Would you think that men or women are more vulnerable to the disease?
     1. Please explain why.
   - Who do you think suffers more from the disease; men or women?
     1. Please explain why.
5. **Coping with schistosomiasis**
   - If you had symptoms related to schistosomiasis, whom would you trust and ask for advice first? (hospital vs traditional healers)
     1. Why?
     2. Do you think there may be differences between men and women in disclosing their schistosomiasis-related symptoms? Please explain.
     3. Do you have any herbs you use for treatment of symptoms related to schistosomiasis? Please describe.
   - Why do you think schistosomiasis remains to be a big problem in your community?
